# Chronic kidney disease in patients with ileal conduit urinary diversion

**DOI:** 10.3892/etm.2012.703

**Published:** 2012-09-11

**Authors:** TOSHIHIDE NAGANUMA, YOSHIAKI TAKEMOTO, SATOSHI MAEDA, TOMOAKI IWAI, NOBUYUKI KUWABARA, TETSUO SHOJI, MIKIO OKAMURA, TATSUYA NAKATANI

**Affiliations:** 1 Departments of Urology and; 2 Endocrinology, Metabolism and Molecular Medicine, Osaka City University Graduate School of Medicine;; 3 Department of Nephrology, Ohno Memorial Hospital, Osaka, Japan

**Keywords:** chronic kidney disease, urinary diversion, ileal conduit, bladder cancer

## Abstract

While renal dysfunction is often observed in patients following urinary diversion due to bladder cancer, there have been few studies on this subject. A cross-sectional study was performed on the renal function of ileal conduit urinary diversion patients and the prevalence and risk factors for chronic kidney disease (CKD) were examined. Patients with ileal conduit urinary diversion (n=102), who were being followed-up as outpatients and who were in stable condition, as well as age- and gender-matched healthy control subjects (n=63) were selected for this study. The prevalence of CKD was compared between the patients and healthy subjects. Next, the clinical factors associated with the presence of CKD were investigated in the patients with ileal conduit diversion using logistic regression analysis. The prevalence of CKD was significantly higher in the patients with ileal conduit diversion compared with the healthy subjects [60 patients (58.8%) vs. 11 healthy subjects (17.5%), P<0.0001]. The mean decrease in the estimated glomerular filtration rate per year of the patients with urinary diversion was 0.95±2.0 ml/min/1.73 m^2^. Multiple logistic regression analysis revealed that the independent and significant factors associated with the presence of CKD were older age and the presence of hypertension, urolithiasis and a past history of hydronephrosis. In conclusion, an increased prevalence of CKD was revealed in the patients with ileal conduit urinary diversion, suggesting the need for better management of hypertension, urolithiasis and hydronephrosis following surgery.

## Introduction

Patients with urinary diversion may exhibit various complications, including upper urinary tract changes (i.e., hydronephrosis or contracted kidney), urinary infections and urolithiasis, and renal dysfunction is often observed (1–5). Over the past decade, the disease concept of chronic kidney disease (CKD) has been advocated and renal dysfunction resulting from urinary diversion should be considered to be an indication of CKD (6–9). It has been reported that the prevalence of renal failure varies widely (10–60%) among cohorts with urinary intestinal diversion (1,5,10), possibly due to inconsistencies in the methods utilized to define renal dysfunction. These studies differ in their definitions of renal failure; renal deterioration and morphological deterioration of the kidney are used synonymously, while the etiology of renal failure is often unknown. In the present study, renal dysfunction was investigated in patients with ileal conduit urinary diversion using the estimated glomerular filtration rate (eGFR), which is a more universal method. A cross-sectional study was performed on the renal function of ileal conduit diversion patients and the prevalence of CKD and risk factors for decreased eGFR was studied.

## Patients and methods

### Study design

A cross-sectional study was performed in patients with ileal conduit urinary diversion.

### Patients

A total of 102 outpatients who had undergone ileal conduit diversion for bladder cancer between April 1979 and March 2011 at Osaka City University Hospital (Osaka, Japan) were studied. The subjects were selected from the total population of patients who had undergone ileal conduit urinary diversion (n=370). Out of the 370 patients, 268 were excluded based on the exclusion criteria which were: subjects with incomplete follow-up (including deceased cases), post-nephrectomy, severe urinary tract infection, chronic glomerulonephritis, chronic inflammatory disease, metastasis or recurrence of bladder cancer or other malignant diseases. Table I shows the characteristics of the 102 patients with ileal conduit diversion.

The control group consisted of 63 age- and gender-matched healthy subjects who voluntarily underwent multiphasic health screening, including a physical examination, electrocardiography, chest X-ray, liver and renal function tests and blood glucose and lipid tests, at Ohno Memorial Hospital between April 2008 and March 2009. Informed consent was obtained from all patients and control volunteers prior to their participation in the study, which was approved by the human ethics committee of Osaka City University Hospital (Osaka, Japan).

### Surgical technique

An ileal segment 15 to 20 cm in length was selected 10 to 15 cm from the ileocecal valve. The ureteral-ileal anastomoses were performed separately by 2 running sutures using the Nesbit technique and were stented with 7- or 8-Fr catheters for 7 to 10 days. The ileal segment was anastomosed to the anterior abdominal wall in a nipple-to-stoma fashion.

### Definition of CKD

In all patients, serum creatinine levels were measured periodically during follow-up and the serum creatinine concentration levels obtained at the patients’ previous visit were used for statistical analysis (mean time from surgery, 9.4±5.5 years; range, 1.8–31.7 years). The eGFR was calculated using the equation of the Japanese Society of Nephrology as follows: 194 x age^−0.287^ x serum creatinine^−1.094^, including a correction factor of 0.739 for females (11). In the present study, CKD was primarily defined as having an eGFR of <60 ml/min/1.73 m^2^ or eGFR of ≥60 ml/min/1.73 m^2^ with proteinuria. Proteinuria was defined as a urinary protein level of ≥1 (∼≥30 mg/dl) in the dipstick test using a spontaneously and freshly voided urine sample, persisting for at least 3 months.

### Risk factors

To evaluate the clinical risk factors, the age and presence or absence of hypertension, diabetes mellitus, dyslipidemia, cardiovascular disease and hydronephrosis were investigated. Hypertension was defined as: i) the administration of antihypertensive agents and/or a history of this disorder; ii) a systolic blood pressure >140 mmHg; or iii) a diastolic blood pressure >90 mmHg. Diabetes mellitus was defined by: i) the administration of insulin or oral antidiabetic agents; or ii) prior diagnosis according to the Report of the Expert Committee on the Diagnosis and Classification of Diabetes Mellitus of the American Diabetes Association (12). Dyslipidemia was defined as a low-density lipoprotein cholesterol level >140 mg/dl, triglyceride >150 mg/dl and high-density lipoprotein cholesterol <40 mg/dl or had undergone medical treatment for hyperlipidemia. Blood samples were obtained following overnight fasting. The diagnosis of pyelonephritis was based on clinical manifestations, body temperature, laboratory test results and imaging data [plain X-ray, ultrasonography and computed tomography (CT)]. Urolithiasis was diagnosed in patients with a history of urolithiasis, as well as those who had no symptoms but were observed to have stone formation in their follow-up CT. Hydronephrosis was diagnosed using ultrasonography, drip infusion pyelography or CT.

### Statistical analysis

The data are expressed as percentages or as the means ± standard deviation (SD), where appropriate. Differences between the groups were examined using the Student’s t-test or Mann-Whitney U test. Changes within each group were evaluated using the paired Student’s t-test. Categorical variables were compared using the Chi-square test. Multiple logistic regression analysis was performed to assess the effect of the variables on the presence of CKD, while dummy variables were used to assess the presence of hypertension, diabetes mellitus, dyslipidemia, urolithiasis, hydronephrosis and pyelonephritis (1 = presence, 0 = absence).

In order to determine the association between hydronephrosis and the presence of CKD, several methods were used to define hydronephrosis in the logistic regression analysis. According to the variables, model 1 contained patients with a past history of hydronephrosis, including prior to surgery, model 2 contained patients with pre-operative hydronephrosis and model 3 contained those with post-operative hydronephrosis. A value of P<0.05 was considered to indicate a statistically significant difference. These tests were performed using the Stat View V Statistical System (SAS Institute Inc., Cary, NC, USA).

## Results

### Demographics and characteristics of the ileal conduit diversion patients with or without CKD and healthy subjects

Table I shows the characteristics of the 102 patients with ileal conduit diversion and the 63 healthy control subjects. The prevalence of CKD was significantly higher in the ileal conduit diversion patients compared with the healthy subjects [60 patients (58.8%) vs. 11 healthy subjects (17.5%), P<0.0001].

The mean age of the patients with CKD was greater than that of the patients without CKD (P<0.0001). The mean post-operative duration of the patients with CKD was longer than that of the patients without CKD (P=0.031).

Hypertension, hydronephrosis, pre-operative hydronephrosis, post-operative hydronephrosis, pyelonephritis and urolithiasis were more prevalent in the patients with CKD (P=0.005, <0.0001, 0.011, 0.001, 0.021, 0.039, respectively). No significant differences were observed in the other factors between the patients with and without CKD. The prevalence of CKD with an eGFR of <60 ml/min/1.73 m^2^ among the patients aged ≥50 years was 47.5% (47/99).

### Temporal changes in eGFR following urinary diversion

**Figure 1:**
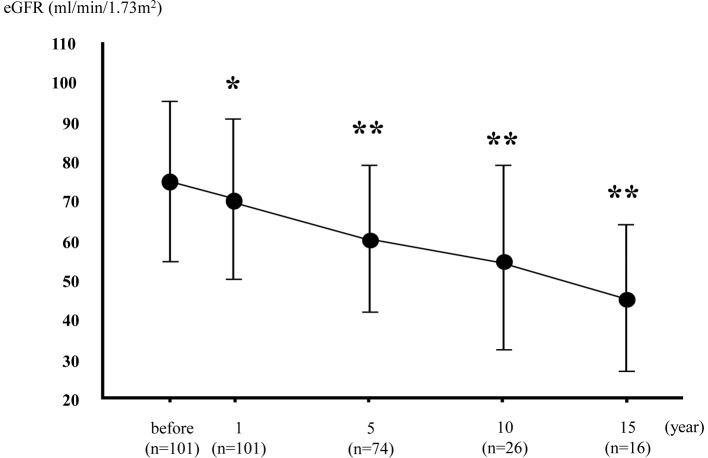
Temporal changes in eGFR following urinary diversion vs. prior to surgery. ^*^P<0.05, ^**^P<0.01. eGFR, estimated glomerular filtration rate.

The mean eGFR values prior to surgery and at 1, 5, 10 and 15 years after surgery for the patients with ileal conduit diversion are shown in Fig. 1 (the cut-off value was 15 years due to the low number patients after 20 years). The mean decrease in eGFR per year for the patients with urinary diversion was 0.95±2.0 ml/min/1.73 m^2^.

### Univariate logistic regression analysis for factors associated with CKD

Univariate logistic regression analysis was performed to examine the correlation between CKD and risk factors in the patients with ileal conduit diversion (Table II). The presence of CKD was significantly associated with older age, longer post-operative duration, the presence of hypertension, a past history of hydronephrosis, post-operative hydronephrosis and urolithiasis.

### Multivariate logistic regression analysis for factors associated with CKD

Multiple logistic regression analysis was performed to examine the factors associated with the presence of CKD, independent of the other factors (Table III). Each multiple model included the unmodifiable basic clinical factors (age, gender, post-operative duration and diabetes mellitus) and the significant factors (hypertension, urolithiasis and hydronephrosis) obtained from the univariate analysis. Models 1 to 3 included a past history of hydronephrosis, pre-operative hydronephrosis and post-operative hydronephrosis, respectively, as variables for hydronephrosis. In these models, older age and the presence of hypertension, urolithiasis, a past history of hydronephrosis and post-operative hydronephrosis were indicated to be independent and significant factors associated with the presence of CKD, whereas the presence of pre-operative hydronephrosis was of borderline significance.

## Discussion

In the present study, we investigated: i) the prevalence of CKD; ii) the temporal changes in post-operative eGFR; and iii) the clinical factors associated with the presence of CKD in patients with ileal conduit urinary diversion. The prevalence of CKD was significantly higher in the ileal conduit diversion patients compared with the healthy subjects. The mean decrease in eGFR per year for the patients with urinary diversion was 0.95±2.0 ml/min/1.73 m^2^. Multiple logistic regression analysis revealed that the independent and significant factors associated with the presence of CKD included older age and the presence of hypertension, urolithiasis and a past history of hydronephrosis.

The overall prevalence of CKD in the ileal conduit diversion patients was 58.8% (60/102), which was significantly higher compared with that of the healthy subjects (17.5%, 11/63; Table I). In the Japanese population aged 50 years or older, the prevalence of CKD with an eGFR less than 60 ml/min/1.73 m^2^ was previously reported to be 18.6% (13). In the present study, among the subjects aged 50 and over, the prevalence of CKD with an eGFR less than 60 ml/min/1.73 m^2^ was 47.5% (47/99). These findings indicated a higher prevalence of CKD in ileal conduit diversion cohorts compared with the age-matched general population, suggesting that ileal conduit urinary diversion may be a risk factor for CKD. Furthermore, the mean yearly decrease in eGFR of the patients was 0.95 ml/min/1.73 m^2^ (Fig. 1), which was approximately 3 times that of previously reported Japanese subjects at 0.36 ml/min/1.73 m^2^ (14). Although it is well-known that renal deterioration is a common complication in ileal conduit diversion patients (1–10), this is the first study to reveal the prevalence of CKD.

In the present study, logistic regression analysis revealed that older age and the presence of hypertension, urolithiasis and a past history of hydronephrosis were the independent and significant factors associated with the presence of CKD (Tables II and III). The results indicating that age and hypertension are clinical risk factors associated with CKD in the ileal conduit urinary diversion patients are consistent with those from previous studies in general populations (15–17). It has also been reported that urolithiasis represents a significant and independent risk factor for CKD in the general population (18), which is consistent with the results of the present study for the patients with the ileal conduit urinary diversion.

Hydronephrosis is a well-known risk factor for CKD, which may cause CKD in ileal conduit diversion patients as well as in cohorts without urinary diversion. The findings of the present study revealed a past history of hydronephrosis in 23.5% of the patients, of which 8.9% exhibited pre-operative hydronephrosis and 14.9% exhibited post-operative hydronephrosis. Singh *et al* (3) reported upper urinary tract dilatation in 34% of patients and Madersbacher *et al* (1) reported that, following ileal conduit diversion, morphological/functional or pre-operative upper urinary tract pathology deterioration occurred in 35/131 (27%) patients. These findings are partially consistent with those of the present study. Multivariate logistic regression analysis indicated that hydronephrosis was a significant risk factor for the presence of CKD in the ileal conduit urinary diversion patients (Tables II and III). Pre-operative hydronephrosis was associated with the presence of CKD to some extent, but post-operative hydronephrosis was more strongly associated (Table III). This may be since renal function may be improved by urinary diversion in renal dysfunction caused by pre-operative urinary obstruction (hydronephrosis) if the kidney function is not impaired as a result of the non-functioning kidney due to chronic obstruction. Ileal conduit urinary diversion is a low pressure reservoir in which outflow is not obstructed, but it may cause hydronephrosis due to the non-physiological attachment of the ureter, anastomotic stricture, stomal stenosis and urolithiasis. Therefore, periodic monitoring following surgery is necessary and when hydronephrosis occurs, it is important to use appropriate monitoring to prevent CKD.

The present study had certain limitations. Firstly, due to the cross-sectional design, the findings do not necessarily indicate causality, but this point may be further elucidated in future longitudinal studies. Secondly, CKD was investigated only in patients with ileal conduit urinary diversion; therefore, further studies are required to elucidate CKD in the patients with other urinary diversion techniques.

In conclusion, we revealed an increased prevalence of CKD in patients with ileal conduit urinary diversion, suggesting the need for improved management of hypertension, urolithiasis and hydronephrosis following surgery. Cancer control is indisputably the most important consideration in the prognosis of patients following total cystectomy, but given the high incidence of CKD, active monitoring of functional/morphological renal changes should be considered to prevent CKD following urinary diversion.

## Figures and Tables

**Table I:** Characteristics of ileal conduit diversion patients with or without CKD and healthy subjects.

Variable	Total (n=102)	Without CKD (n=42)	With CKD (n=60)	P-value^a^	Healthy subjects (n=63)
Age (years), mean ± SD	69.1±8.9	65.1±9.3	72.5±6.8	<0.0001	68.0±8.1
Gender (males), n (%)	79 (77.5)	32 (76.2)	47 (78.3)	NS	43 (68.3)
Hypertension, n (%)	37 (36.3)	8 (19.0)	29 (48.3)	0.005	16 (25.4)
Diabetes mellitus, n (%)	16 (15.7)	5 (11.9)	10 (16.7)	NS	6 (9.5)
Dyslipidemia, n (%)	18 (17.6)	4 (9.5)	14 (23.3)	NS	22 (34.9)
Post-operative duration (years), mean ± SD	9.4±5.5	7.9±3.1	10.3±6.6	0.031	-
Hydronephrosis, n (%)	24 (23.5)	2 (4.8)	22 (36.7)	<0.0001	-
Pre-operative hydronephrosis, n (%)	9 (8.8)	1 (2.4)	8 (13.3)	0.011	-
Post-operative hydronephrosis, n (%)	15 (14.7)	1 (2.4)	14 (23.3)	0.001	-
Past history of pyelonephritis, n (%)	33 (32.4)	7 (16.7)	24 (40.0)	0.021	-
Urolithiasis, n (%)	14 (13.7)	2 (4.8)	12 (20.0)	0.039	-
eGFR (ml/min/1.73 m^2^), mean ± SD	62.3±22.0	78.0±15.7	50.6±17.3	<0.0001	78.4±22.1
Proteinuria, n (%)	26 (25.5)	0 (0)	26 (43.3)	<0.0001	2 (3.2)
CKD, n (%)	60 (58.8)	-	-	-	11 (17.5)

CKD, chronic kidney disease; eGFR, estimated glomerular filtration rate.

a Without CKD vs. with CKD.

**Table II:** Univariate logistic regression analysis of factors associated with CKD in patients with ileal conduit diversion.

Variable	Unit increase	OR (95% CI)	P-value
Age	1 year	1.14 (1.07–1.22)	<0.0001
Gender (males vs. females)		1.13 (0.44–2.89)	0.888
Post-operative durations	1 year	1.10 (1.01–1.20)	0.039
Hypertension (presence vs. absence)		3.98 (1.58–9.99)	0.003
Diabetes mellitus (presence vs. absence)		1.20 (0.40–3.60)	0.745
Dyslipidemia (presence vs. absence)		2.89 (0.88–9.52)	0.081
Past history of hydronephrosis (presence vs. absence)		11.58 (2.55–52.64)	0.0015
Pre-operative hydronephrosis (presence vs. absence)		6.31 (0.76–52.50)	0.089
Post-operative hydronephrosis (presence vs. absence)		9.94 (2.11–46.85)	0.004
Past history of pyelonephritis (presence vs. absence)		2.44 (0.99–6.01)	0.052
Urolithiasis (presence vs. absence)		5.00 (1.06–23.67)	0.043

Significant factors associated with chronic kidney disease (CKD) were analyzed, including age, gender (female = 0, male = 1), post-operative duration, hypertension (absent = 0, present = 1), diabetes mellitus (absent = 0, present = 1), dyslipidemia (absent = 0, present = 1), a past history of hydronephrosis (absent = 0, present = 1), pre-operative hydronephrosis (absent = 0, present = 1), post-operative hydronephrosis (absent = 0, present = 1), past history of pyelonephritis (absent = 0, present = 1) and urolithiasis (absent = 0, present = 1). OR, odds ratio; CI, confidence interval.

**Table III:** Multiple logistic regression analysis of factors associated with CKD in patients with ileal conduit diversion.

Variable	Unit increase	Model 1 OR (95% CI)	Model 2 OR (95% CI)	Model 3 OR (95% CI)
Age	1 year	1.16 (1.07–1.26)^c^	1.16 (1.07–1.25)^c^	1.17 (1.08–1.27)^c^
Gender (males vs. females)		0.89 (0.23–3.49)	0.92 (0.26–3.31)	0.85 (0.23–3.08)
Post operative durations	1 year	1.06 (0.94–1.19)	1.05 (0.94-.18)	1.06 (0.95–1.19)
Hypertension (presence vs. absence)		4.03 (1.20–13.56)^a^	4.70 (1.46–15.03)^b^	4.26 (1.31–13.78)^a^
Diabetes mellitus (presence vs. absence)		0.63 (0.16–2.49)	0.65 (0.18–2.37)	0.62 (0.17–2.32)
Urolithiasis (presence vs. absence)		15.76 (1.93–129.04)^a^	13.45 (1.83–98.69)^a^	14.35 (1.73–119.09)^a^
Past history of hydronephrosis (presence vs. absence)		21.85 (2.22–214.83)^b^	-	-
Pre-operative hydronephrosis (presence vs. absence)		-	32.14 (0.38–2720.88)	-
Post-operative hydronephrosis (presence vs. absence)		-	-	15.41 (1.16–204.98)^a^

Significant factors associated with chronic kidney disease (CKD) were analyzed, including age, gender (female = 0, male = 1), post-operative duration, hypertension (absent = 0, present = 1), diabetes mellitus (absent = 0, present = 1), urolithiasis (absent = 0, present = 1), a past history of hydronephrosis (absent = 0, present = 1), pre-operative hydronephrosis (absent = 0, present = 1), post-operative hydronephrosis (absent = 0, present = 1).

a P<0.05,

b P<0.01,

c P<0.001. The R^2^ values were 0.38 for model 1, 0.34 for model 2 and 0.34 for model 3 (all P<0.0001). OR, odds ratio; CI, confidence interval.
